# Endotype Classification of Nonsteroidal Anti‐Inflammatory Drug‐Exacerbated Respiratory Disease According to Chronic Rhinosinusitis Comorbidity

**DOI:** 10.1111/cea.70341

**Published:** 2026-05-19

**Authors:** Soyoon Sim, Jieun Seo, Junhyuk Chang, Yoo Seob Shin, Rae Woong Park, Hae‐Sim Park

**Affiliations:** ^1^ Department of Allergy and Clinical Immunology Ajou University School of Medicine Suwon Korea; ^2^ Department of Biomedical Sciences Ajou University Graduate School of Medicine Suwon Korea; ^3^ Department of Biomedical Informatics Ajou University School of Medicine Suwon Korea

## Abstract

- CRSw/sNP exacerbates T2/eosinophilic inflammation and lung function decline, worsening clinical outcomes in patients with nonsteroidal anti‐inflammatory drug (NSAID)‐exacerbated respiratory disease (N‐ERD).
- Endotype classification of N‐ERD according to CRSw/sNP comorbidity facilitates prognostic assessment and precision medicine.

To the Editor,

Nonsteroidal anti‐inflammatory drug (NSAID)‐exacerbated respiratory disease (N‐ERD) is a chronic inflammatory disease of the upper and lower airways characterized by moderate‐to‐severe asthma and a high prevalence of chronic rhinosinusitis with recurrent nasal polyps (CRSwNP). Patients with N‐ERD present more severe clinical outcomes, such as frequent asthma exacerbations (AEs) and emergency department (ED) visit/hospitalization rates than patients with NSAID‐tolerant asthma (NTA), remaining poorly controlled even on higher doses of inhaled or systemic corticosteroids (SCSs) [1].

N‐ERD exhibits considerable heterogeneity according to the presence of comorbid disease, leading to diagnostic delay and disease burden. Thus, phenotype/endotype classification is crucial for early diagnosis and individual treatment. Notably, CRSwNP can worsen disease severity and clinical outcomes, driving lung function decline in patients with N‐ERD [2, 3]. This study aimed to evaluate longitudinal changes in clinical outcomes and inflammatory profiles according to CRSw/sNP comorbidity in a large cohort of patients with N‐ERD compared with NTA patients during a 10‐year period.

In this retrospective study, medical records from Ajou University Medical Center (Suwon, South Korea) were utilized. A total of 467 N‐ERD and 1401 patients with NTA were enrolled after 1:3 propensity score matching and classified into four endotypes according to comorbid conditions: Group 1, N‐ERD with CRSw/sNP; Group 2, N‐ERD without CRS or NP; Group 3, NTA with CRSw/sNP; and Group 4, NTA without CRS or NP. Detailed methodology is available in the Open Science Framework repository (https://doi.org/10.17605/OSF.IO/A2986).

When the baseline clinical characteristics and changes in long‐term clinical outcomes were compared between patients with N‐ERD and NTA, the prevalences of severe asthma (SA), CRS and NPs, blood eosinophil counts (BECs), and SCS use were significantly higher in patients with N‐ERD than in patients with NTA (*p* < 0.05 for all). Furthermore, patients with N‐ERD had more frequent AEs and ED visits/hospitalizations and higher required doses of intravenous (IV)/SCS than patients with NTA (*p* < 0.001 for all). The values of forced expiratory volume in 1 s (FEV_1_, %) decreased in both groups, showing persistently lower levels in patients with N‐ERD than in patients with NTA but did not significantly differ. Conversely, fractional exhaled nitric oxide (FeNO) values increased in both groups, maintaining significantly higher in patients with N‐ERD than in patients with NTA (*p* = 0.003). BECs steadily decreased in patients with NTA but remained higher in patients with N‐ERD throughout the study period (*p* = 0.001). Sputum eosinophils (%) were higher in patients with N‐ERD but not statistically significant. Additional results are described in https://doi.org/10.17605/OSF.IO/A2986.

When patients with N‐ERD and NTA were classified according to CRSw/sNP comorbidity, Group 1 showed lower FEV_1_ and FEV_1_/forced vital capacity (FVC, %) than Group 3 (*p* = 0.006; *p* = 0.007, respectively) but higher FeNO than Groups 2 and 4 (*p* = 0.001; *p* = 0.002, respectively). Furthermore, Group 1 had the highest rates of CRSw/sNP comorbidities and SCS uses among the groups (*p* < 0.05 for all). Moreover, higher blood/sputum eosinophils but lower sputum neutrophils were noted in Group 1 than in Group 2 (*p* < 0.05 for all; Table 1). Long‐term clinical outcome analyses demonstrated that Group 1 had more frequent AEs and higher IV/SCS uses than the three other groups (*p* < 0.05 for all), and more frequent ED visit/hospitalization than Groups 3 and 4 (*p* < 0.001 for all). FEV_1_ progressively decreased in Groups 1 and 3 but increased in Groups 2 and 4, showing persistently lower levels in Group 1 than in Groups 2 and 4 (*p* = 0.005; *p* = 0.026, respectively). FeNO increased and remained higher in Group 1 but did not significantly differ from the three other groups. BECs were higher in Groups 1 and 3 than in Groups 2 and 4 at the baseline; they rapidly declined in Group 3 but remained constantly higher in Group 1 during the follow‐up period (*p* < 0.001). Sputum eosinophils remained highest in Group 1 among the other groups but did not significantly differ. Additional analyses are detailed in https://doi.org/10.17605/OSF.IO/A2986.

**TABLE 1:** Comparison of baseline clinical characteristics according to CRSw/sNP comorbidity in patients with N‐ERD and NTA.

Variable	N‐ERD		NTA		*p* ^a^	*p* ^b^	*p* ^c^	*p* ^d^	*p* ^e^
	Group 1 (*n* = 358)	Group 2 (*n* = 109)	Group 3 (*n* = 417)	Group 4 (*n* = 984)					
Female	246 (68.7)	80 (73.4)	295 (70.7)	687 (69.8)	0.800	0.416	0.593	0.784	0.778
Age (years)	45.2 ± 13.8	45.8 ± 17.8	45.1 ± 13.6	45.2 ± 15.4	0.980	0.756	0.921	0.966	0.941
BMI (kg/m^2^)	23.8 ± 2.5	17.5 ± NA	22.5 ± 2.9	23.0 ± 2.8	0.185	—	0.285	0.466	0.524
Pulmonary functions									
FVC (%)	89.4 ± 15.6	93.8 ± 16.9	91.2 ± 16.4	88.6 ± 17.2	0.200	0.157	0.481	0.706	0.190
FEV_1_ (%)	89.8 ± 18.6	93.5 ± 21.4	94.2 ± 18.5	91.4 ± 20.9	0.061	0.131	0.006	0.259	0.056
FEV_1_/FVC (%)	80.6 ± 9.7	83.3 ± 15.4	83.2 ± 8.9	81.6 ± 12.9	0.125	0.196	0.007	0.283	0.066
FeNO (ppb)	52.5 ± 47.7	33.7 ± 24.3	49.0 ± 50.7	36.0 ± 40.2	0.002	0.001	0.585	0.002	0.019
Asthma‐related variables									
Severe asthma, *n* (%)	52 (14.5)	7 (6.4)	26 (6.2)	60 (6.1)	< 0.001	0.036	< 0.001	< 0.001	1.000
Nasal polyp, *n* (%)	115 (32.1)	0 (0)	38 (9.1)	0 (0)	0.000	< 0.001	< 0.001	< 0.001	< 0.001
BEC (cells/μL)	517.4 ± 561.6	233.7 ± 244.5	444.7 ± 688.3	298.7 ± 415.2	< 0.001	< 0.001	0.121	< 0.001	< 0.001
Sputum eos (%)	40.4 ± 35.3	23.4 ± 26.1	42.7 ± 32.6	34.2 ± 32.6	0.023	0.044	0.564	0.095	0.021
Sputum neu (%)	57.7 ± 33.4	75.7 ± 23.1	59.9 ± 31.3	66.2 ± 29.9	< 0.001	< 0.001	0.496	0.002	0.016
Serum total IgE (KU/L)	374.7 ± 580.8	437.1 ± 558.8	401.9 ± 637.9	395.4 ± 687.0	0.879	0.384	0.583	0.641	0.888
SCSs, *n* (%)	277 (77.4)	67 (61.5)	225 (54.0)	533 (54.2)	< 0.001	0.001	< 0.001	< 0.001	0.989

*Note:* Continuous values are presented as mean ± SD and categorical variables are presented as number (%).

Abbreviations: BMI, body mass index; eos, eosinophil; FeNO, fractional exhaled nitric oxide; FEV_1_, forced expiratory volume in 1 s; FVC, forced vital capacity; N‐ERD, nonsteroidal anti‐inflammatory drug‐exacerbated respiratory disease; neu, neutrophil; NP, nasal polyps; NTA, nonsteroidal anti‐inflammatory drug‐tolerant asthma; SCS, systemic corticosteroids.

^a^ Among 4 groups.

^b^ Group 1 versus 2.

^c^ Group 1 versus 3.

^d^ Group 1 versus 4.

^e^ Group 3 versus 4.

In the present study, four distinct endotypes comparing patients with N‐ERD and NTA according to CRSw/sNP comorbidity was demonstrated by analysing 10‐year follow‐up data in a real‐world setting. Moreover, patients with N‐ERD with CRSw/sNP presented higher T2 signatures and progressive lung function decline, causing worse clinical outcomes than those without CRS or NP or NTA with CRSw/sNP [2, 4]. Notably, despite sharing CRSw/sNP, BEC progressively declined in NTA, reflecting the favourable treatment responses. However, patients with N‐ERD maintained persistently high BECs, indicating that T2/eosinophilic inflammation in N‐ERD is driven not only by local CRSw/sNP but also by systemic metabolic dysfunction, including leukotriene overproduction, resistant to conventional therapies [5, 6]. Thus, N‐ERD with CRSw/sNP represents a high‐risk endotype accompanying increased asthma severity and poor disease control, requiring early intervention of T2 biologics to achieve disease control or clinical remission [7, 8, 9].

This study has limitations. First, only the presence of CRSw/sNP was applied for endotype classification of N‐ERD. Second, as a single‐center retrospective study, residual confounding from clinically significant variables—such as smoking status, environmental exposures, allergen sensitization, medication adherence and prior sinus surgery—was not included. Further multi‐center prospective and replication studies considering other comorbidities are needed in larger cohorts for more detailed endotype classification.

In conclusion, we suggested the long‐term, longitudinal characteristics of the distinct phenotype/endotype in N‐ERD (which were distinguished from those of NTA) in a Korean cohort tracking 467 N‐ERD and 1401 NTA patients during a 10‐year period. Classifying patients with N‐ERD according to CRSw/sNP comorbidity is helpful for predicting long‐term clinical outcomes in real‐world practice, facilitating precise prognostic assessment and tailored therapeutic strategies with T2 biologics.

## Author Contributions

Soyoon Sim – investigation, writing – original draft. Jieun Seo – visualization, writing – original draft. Junhyuk Chang – formal analysis. Yoo Seob Shin and Rae Woong Park – project administration. Hae‐Sim Park – conceptualization, supervision, funding acquisition, writing – review and editing.

## Funding

This work was supported by the Ministry of Health and Welfare (RS‐2024‐00439277).

## Conflicts of Interest

The authors declare no conflicts of interest.

## Data Availability

The data that support the findings of this study are available from the corresponding author upon reasonable request.
